# Immunological characterization and function analysis of L-type lectin from spotted knifejaw, *Oplegnathus punctatus*

**DOI:** 10.3389/fimmu.2022.993777

**Published:** 2022-09-26

**Authors:** Jinxiang Liu, Xiaobing Liu, Zhigang Wang, Quanqi Zhang

**Affiliations:** ^1^ MOE Key Laboratory of Marine Genetics and Breeding, College of Marine Life Sciences, Ocean University of China, Qingdao, China; ^2^ Laboratory for Marine Fisheries Science and Food Production Processes, Qingdao National Laboratory for Marine Science and Technology, Qingdao, China; ^3^ Key Laboratory of Tropical Aquatic Germplasm of Hainan Province, Sanya Oceanographic Institution, Ocean University of China, Sanya, China; ^4^ Hainan Yazhou Bay Seed Laboratory, Sanya, China

**Keywords:** lily-type lectin, agglutination, hemagglutination, innate immune, *Oplegnathus punctatus*

## Abstract

Lily-type lectin (LTL) plays significant roles in innate immune response against pathogen infection. LTL in animals and plants has received widespread attention. In the present study, an LTL (OppLTL) was identified from spotted knifejaw *Oplegnathus punctatus*. The OppLTL encoded a typical Ca^2+^-dependent carbohydrate-binding protein containing a CRD domain. The qRT-PCR showed that it was mainly expressed in the gill and was significantly upregulated after *Vibrio anguillarum* challenge. The agglutination analysis showed that the recombinant OppLTL could bind and agglutinate Gram-negative and Gram-positive bacteria in a Ca^2+^-dependent manner. However, the binding activity was different. Meanwhile, the recombinant OppLTL could hemagglutinate mammalian and teleost erythrocytes. Subcellular localization revealed that OppLTL was mainly detected in the cytoplasm of HEK293T cells. The dual-luciferase analysis revealed that OppLTL could inhibit the activity of the NF-κB signal pathway in HEK293T cells after OppLTL overexpression. These findings collectively demonstrated that OppLTL could be involved in host innate immune response and defense against bacterial infection in spotted knifejaw.

## Introduction

Lectins are a group of sugar-binding proteins, which are distributed in living organisms from viruses to humans (1). Animal lectins contain one or several carbohydrate recognition domains (CRDs). On the basis of their structures, motif types, binding specificities, and calcium dependency, they are classified into several main families such as C-, L-, P-, I-, R-, F-, and S-type lectins, calnexin, pentraxins, and C-reactive protein (2). Lectins act as a mediator of self- and non-self-recognition and play a significant role in cellular functions, including cell communication, proliferation, phagocytosis, signal transduction, metastasis, and apoptosis (3). Lectins have diverse functions in different organisms. Lectins belong to pattern recognition receptor (PRR) class and sugar-binding proteins, and they could recognize the exposed carbohydrates on the surface of potential pathogenic microbes. Lectins also act as innate immunity factors to induce agglutination, immobilization, and complement-mediated opsonization and killing of pathogens. In pathogens, lectins are used to recognize host cell surface glycans as colonization factors (4–6).

Lily-type lectin (LTL) was first identified in the skin mucus of pufferfish (*Fugu rubripes*), which intriguingly shared homology with D-mannose-binding lectin in plants. It was also named pufflectin (7). Aside from pufferfish, fish LTLs had been identified from grass pufferfish (*Takifugu niphobles*), snakehead murrel (*Channa striata*), turbot (*Scophthalmus maximus*), rock bream (*Oplegnathus fasciatus*), large yellow croaker (*Larimichthys crocea*), orange-spotted grouper (*Epinephelus coioides*), tongue sole (*Cynoglossus semilaevis*), and black rockfish (*Sebastes schlegelii*) (8–14). LTLs in teleost were characterized by a threefold internal repeat and two conserved mannose-binding motifs of QXDXNXVXY. The motif could interact with ligands (15). Lectins are involved in innate immune response. They can defend against pathogens through bacterial agglutination binding and coagulation (16–21). A previous study on lectins in spotted knifejaw found that C-type lectin was upregulated after a pathogen infection, and the recombinant C-type lectin could inhibit the activity of the NF-κB signal pathway (22).

Spotted knifejaw is a cultured fish species in China. Studies on the potential functions of these lectins in immune response are scarce. In this study, the LTL of spotted knifejaw was identified, and its function was explored. The expression pattern was detected after pathogen infection. Furthermore, bacterial agglutination, binding, and coagulation activity analyses were performed to verify the innate immune function as a PRR. Our findings might expand the understanding of the anti-bacterial immune mechanism of spotted knifejaw, especially the anti-bacterial mechanism of lectins, by comparing the expression profiles and biological properties of LTL.

## Materials and methods

### Fish, cell line, and bacterium

The experimental spotted knifejaws (6 months old) were collected from Laizhou MingBo Aquatic Co., Ltd. (Shandong Province, China). They were acclimated in aerated seawater for 7 days at 25°C before the bacterial challenge. The absence of *V. anguillarum* and *Edwardsiella tarda* was tested by PCR amplifications using species-specific primers before the experiment (**Table 1**). The tissues for qRT-PCR were collected from three individuals. The samples were snap-frozen in liquid nitrogen and stored at −80°C. The pathogens, including *Bacillus subtilis* (LB medium, 37°C), *Staphylococcus aureus* (LB medium, 37°C), *Escherichia coli* (LB medium, 37°C), *Vibrio anguillarum* (LB medium, 37°C), *E. tarda* (LB medium, 28°C), and *Aeromonas hydrophila* (TSB medium, 28°C), were acquired from the Key Laboratory of Microbial Oceanography, Ocean University of China. The HEK293T cell line was cultured in DMEM supplemented with 10% fetal bovine serum and 4 mM L-glutamine at 37°C in a humidified atmosphere containing 95% air and 5% CO_2_.

**Table 1:** List of the primer sequences used in the study.

Primers	Sequence (5′-3′)	Tm (°C)	Usage
*OppLTL*-RT-Fw	GTGGGCTTCAGACACTTG	60	qRT-PCR
*OppLTL*-RT-Rv	GTCATCGGTCAGTTGAAGAC	60	qRT-PCR
*OppLTL*-Fw	CGCGGATCCATGAGTAAGAACTACTTGTCCAGA	55	plasmid construction
*OppLTL*-Rv	CCGCTCGAGTCATTTCTTTGAACCTTTGG	55	plasmid construction
*OppLTL*-Fw2	CCGCTCGAGATGAGTAAGAACTACTTGTC	55	plasmid construction
*OppLTL*-Rv2	TCCCCCGCGGTTTCTTTGAACCTTTGGAGT	55	plasmid construction
*β-actin*-Fw	GGTCTGTGATGCCCTTAGATGTC	60	qRT-PCR
*β-actin*-Rv	AGTGGGGTTCAGCGGGTTAC	60	qRT-PCR
*Va*-Fw	CTGCTGATGTCCACCCTACA	60	*V. anguillarum* detection
*Va*-Rv	CGCTGTAAATTCCAGCCC	60	*V. anguillarum* detection
*Et*-Fw	GCAGATGTATCATGGT	55	*E. tarda* detection
*Et*-Rv	TAATGCGGTTGGTATCCACA	55	*E. tarda* detection

### Fish challenge and sample collection

A total of 57 6-month-old healthy individuals (~40 g) were used for the challenge experiment. The fish were randomly divided into three groups denoted as the blank control group, control group, and treatment group, which included 3, 27, and 27 individuals, respectively. Each group was reared in three independent tanks containing approximately 36 L of aerated seawater. The blank control group did not receive any treatment. The control group was injected with 100 μl of phosphate-buffered saline [phosphate-buffered saline (PBS), pH = 7.2]. The treatment group was intraperitoneally injected with *V. anguillarum* suspension (5 × 10^5^ CFU/ml in PBS). Three individuals were collected from the blank control group at 0 h post-injection. At 2, 4, 8, 12, 24, 48, 72, 96, and 120 h post-injection, three individuals were randomly collected from the control and treatment groups, respectively. Tissues were collected and snap-frozen in liquid nitrogen and stored at −80°C until RNA extraction.

### Total RNA extraction and cDNA synthesis

Total RNA was extracted using TRIzol Reagent (Invitrogen, USA) in accordance with the manufacturer’s protocol. It was treated with RNase-free DNase I (TaKaRa, Dalian, China) to degrade genomic DNA. Reverse transcription and cDNA synthesis were performed with 1 μg of total RNA and random primers using the Reverse Transcriptase M-MLV Kit (TaKaRa, Dalian, China) in accordance with the manufacturer’s instructions. The quality and quantity were evaluated *via* 1.5% agarose gel electrophoresis and spectrophotometry using NanoPhotometer Pearl (Implen GmbH, Germany).

### Sequence identification and analysis

To identify the *OppLTL* gene, the available sequences of teleost *LTL* cDNA were acquired from NCBI and Ensembl. The retrieved sequences were used as query sequences in BLAST searches. The mRNA sequence of the *OppLTL* gene was identified using tBLASTn analysis from spotted knifejaw transcriptome previously sequenced by our laboratory. The theoretical isoelectric point (pI) and the molecular weight (MW) were calculated using online ExPASy Compute pI/Mw tool. Protein domains were predicted using the Simple Modular Architecture Research Tool. The 3-D structure was predicted by I-TASSER. Model-2 was selected as the optimal model based on the C-score value. Ramachandran plot analysis was used to test the rationality of the structure. PyMOL was used for visualization. The sequence alignment of *LTL* was based on its predicted peptide sequences using Clustal X with default parameters (23). A phylogenetic tree was constructed based on the deduced amino acid sequences using MEGA X, and 1,000 bootstraps were selected to evaluate reliability.

### qRT-PCR

The specific primer pair (*OppLTL*-RT-Fw/Rv, **Table 1**) was designed based on the characteristics of *OppLTL*. During the pre-experiment, standard curves were established, and efficiency values were calculated. Product specificity was ensured through a melting curve analysis which consisted of 40 cycles. Furthermore, qRT-PCR was performed using the SYBR Premix Ex TaqII (TaKaRa, Dalian, China) on a LightCycler 480 system. *β-actin* was selected as the reference gene (22). The relative expression levels of the target gene were calculated as the ratio of the target gene copy number to the *β-actin* copy number. The amplification conditions were as follows: 95°C for 60 s and then 45 cycles of 95°C for 5 s and 60°C for 30 s. Three biological replicate RNA samples from healthy and challenged tissues were analyzed for gene expression. The relative quantities were calculated using the 2^-ΔΔCt^ comparative Ct method.

### Plasmid construction and purification of OppLTL protein

For the expression protein, the ORF of *OppLTL* was amplified using a specific primer (*OppLTL*-Fw1/Rv1) containing the restriction enzyme sites *BamHI* and *XhoI*. The PCR product was subcloned into the pET-32a (+) vector to construct the expression vector pET-32a-OppLTL. The recombinant plasmid was validated by double enzyme digestion and sequencing. The expression plasmid was transformed into *E. coli* BL21 (DE3) cells, and the positive clone was screened by PCR and sequencing. Isopropyl β-D-thiogalactopyranoside (IPTG) was used to induce recombinant OppLTL (rOppLTL) at a final concentration of 0.5 mM at 37°C for 8 h. The expression, purification, and refolding of rOppLTL were performed according to the methods of Qu et al. (24). Meanwhile, recombinant His-tag (rTRX) was expressed and purified as control. The purified proteins were analyzed by 12% SDS-PAGE and stained with Coomassie Brilliant Blue R-250. The concentrations of recombinant proteins were determined by BCA Protein Assay Kit (Tiangen Biotech, China).

### Western blot analysis

The proteins on the gels were electroblotted onto a polyvinylidene fluoride membrane (Amersham, USA) using a semi-dry technique (Bio-Rad, USA). After blocking with PBS, containing 5% nonfat milk for 1 h at room temperature, the membranes were incubated with anti-His-tag rabbit antibody (CWBIO, China) diluted 1:4,000 with tris-buffered saline (TBS) containing 5% nonfat milk at 4°C overnight. After washing five times with TBS containing 0.1% Tween-20 (TBST), the membranes were incubated with horseradish peroxidase-conjugated goat anti-rabbit IgG Ab (CWBIO, China) diluted 1:8,000 with TBS containing 5% nonfat milk at room temperature for 40 min. The bands were visualized using DAB kit (CWBIO, China) according to the manufacturer’s instruction. Subsequently, digital images were captured with the Bio-Rad ChemiDoc MP Imaging System.

### Microorganism binding assay

Four Gram-negative bacteria (*E. coli*, *V. anguillarum*, *E. tarda*, and *A. hydrophila*), and two Gram-positive bacteria (*S. aureus* and *B. subtilis*) were used to measure the bacterial binding activity of rOppLTL. The microorganisms were collected by centrifugation at 6,000 rpm for 5 min at the mid-logarithmic phase and then washed twice with TBS. During the binding assay, the microorganisms were resuspended with TBS, giving a density of 1 × 10^8^ cells/ml. Next, 200 μl aliquots of bacterial suspensions were mixed with 100 μl of rOppLTL (200 μg/ml). The experiment was divided into three groups, including OppLTL, OppLTL and CaCl_2_, and OppLTL and CaCl_2_ added with EDTA for chelating Ca^2+^. The mixtures were incubated at 24°C for 1 h in the presence or absence of 10 mM CaCl_2_ and centrifuged at 6,000 rpm for 5 min. The pellets of microorganism were washed three times with TBS and resuspended in 100 μl TBS. The elution and final pellets of microorganisms were analyzed by 12% SDS-PAGE, and the binding activity was determined by western blot analysis.

### Assay for the binding of polysaccharides

Lipopolysaccharide (LPS) and peptidoglycan (PGN) were used to detect the binding ability of rOppLTL by ELISA. They were dissolved in water to achieve a concentration of 40 μg/ml, and 50 μl of LPS or PGN was applied to each well of the 96-well microplate which was air-dried at 25°C overnight. The plates were incubated at 60°C for 30 min to fix the ligands. Then, each well was blocked with 100 μl of 10 mg/ml bovine serum albumin (BSA) in TBS at 37°C for 3 h. After washing four times with TBST, a total of 100 μl TBS containing 0.1 mg/ml BSA and different concentrations of rOppLTL were added into each well in the presence of 10 mM CaCl_2_, and the wells were incubated at room temperature for 3 h. The wells were washed as aforementioned and then incubated with 100 μl of rabbit anti-His-tag antibody (CWBIO, China) diluted 1:5,000 with 1 mg/ml BSA in TBS at 37°C for 1 h. After washing four times with TBST, each well was incubated with 100 μl of peroxidase-conjugated goat anti-rabbit IgG Ab (CWBIO, China) diluted 1:8,000 with 1 mg/ml BSA in TBS at room temperature for 1 h. The plate was washed four times as previously described, and color was developed by adding 100 μl TMB (Solarbio, China). The reaction was stopped by adding 2 M H_2_SO_4_, and the absorbance was read at 450 nm. TBS was used in the negative control instead of recombinant protein. The assay was repeated thrice.

### Microorganism agglutination and hemagglutination assay

Six microorganisms were used to measure the agglutination activity of rOppLTL according to the method of Qu et al. (24). They were cultured and collected. Then, they were washed three times with PBS and resuspended in TBS, yielding a density of 2 × 10^8^ cells/ml. Aliquots of 25 μl of bacterial suspensions were mixed with 25 μl of rOppLTL (200 μg/ml) or rTRX (control) in the presence or absence of 10 mM CaCl_2_. Then, they were incubated at 37°C for 1 h and observed under a microscope (Nikon, Japan). All experiments were performed in triplicate.

The hemagglutination assay was performed according to the method described by Zhang et al. (25). Briefly, peripheral blood was collected, and then erythrocytes were derived *via* centrifugation at 3,000 rpm for 5 min. After washing four times with TBS, these erythrocytes were resuspended with 2% TBS and diluted to equal concentrations. Next, 25 μl of pretreatment half-diluted rOppCTL (500 μg/ml) and 25 μl cell suspension were mixed and cultured for 1 h in a 96-well plate in the presence or absence of 10 mM CaCl_2_. Then, hemagglutination results were obtained.

### Transfection and subcellular localization

The vector pEGFP-OppLTL was constructed by pEGFP-N1 to explore the localization of OppLTL in HEK293T cell. The specific primers (*OppLTL*-Fw2/Rv2) containing the restriction enzyme sites *XhoI* and *SacII* were used to amplify the ORF of *OppLTL*. Before the experiment, a total of 5 × 10^5^ cells were seeded into 12-well plates and cultured for 24 h. Then, the pEGFP-OppLTL vector was transfected into HEK293T cells by Lipofectamine™ 3000 Transfection Reagent (Thermo Fisher Scientific, USA) according to the manufacturer’s instructions. The control group was transfected with an equal amount of pEGFP-N1.

The cells were seeded on coverslips in six-well plates for overnight growth. Then, they were washed twice with PBS, fixed with 4% paraformaldehyde for 10 min, and permeabilized with 0.5% Triton X-100 for 20 min at room temperature. The coverslips were washed with PBS and incubated overnight with the primary antibody (Abcam, ab6556) at 4°C. After washing with PBS, the coverslips were covered with the secondary antibody (FITC-conjugated rabbit anti-GFP) for 1 h at room temperature. After rinsing with PBS, the cells were stained with 2 μg/ml of DAPI in PBS for 10 min. The cells were rinsed twice with PBS after staining and visualized with a confocal microscope (Nikon, Japan).

### Dual-luciferase reporter assay

The ORF of OppLTL containing the restriction enzyme sites *XhoI* and *SacII* were amplified and inserted into the pEGFP-N1 vector to construct the expression vector. Before the experiment, a total of 5 × 10^5^ HEK293T cells were seeded into 24-well plates and cultured for 24 h. Furthermore, the cells were transiently co-transfected with NF-κB reporter vectors (100 ng/well), pRL-TK vectors (20 ng/well), and targeted recombined vectors (500 ng/well) by Lipofectamine™ 3000 Transfection Reagent (Thermo Fisher Scientific, USA). The Renilla luciferase pRL-Tk vector (Promega, USA) was used as an internal control. At 12, 24, and 48 h post-transfection, the cells were washed with PBS and analyzed for Luc activity using the luciferase assay system (Promega, USA). Each experiment was repeated six times. To determine the effect of the recombined vector of OppLTL concentration on the activation level of the NF-κB signal pathway, we set up a series of concentration gradients of OppLTL (100, 200, and 500 ng/well). The cells were lysed at 48 h post-transfection and measured using the luciferase reporter assay system. The relative luciferase values were calculated by normalizing the firefly luciferase activity on the basis of Renilla luciferase activity. The experimental results are expressed as fold stimulation changes relative to the empty vector.

### Statistical analysis

The data were statistically analyzed using two-way ANOVA followed by LSD test by using SPSS 20.0 (IBM, USA). *P <*0.05 indicated a statistical significance between groups. Data are presented as mean ± SD.

## Results

### Characterization of *OppLTL* cDNA

**Figure 1:**
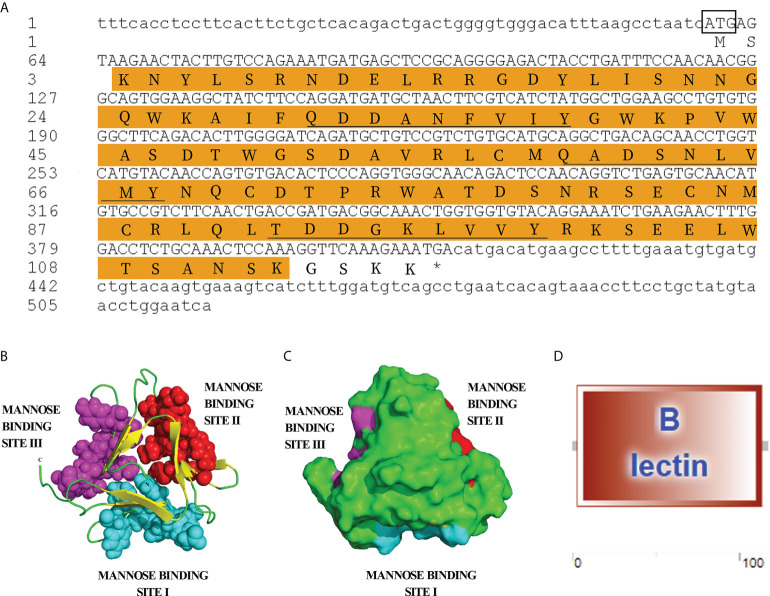
Nucleotide and protein structure of OppLTL. **(A)** Nucleotide sequence of OppLTL cDNA and deduced amino acid sequence. The start codon is boxed, and the stop codon is marked with an asterisk. The shaded part represents B-lectin domain, and the three D-mannose binding sites are underlined. **(B)** 3-D structure of OppLTL. Random coils are marked with green color, and yellow parts represent β-sheets. The D-mannose binding sites-1, -2, and -3 are represented in cyan-, red-, and purple-colored ball structure, respectively. **(C)** Surface view of OppLTL. **(D)** Predicted domain of OppLTL.

The cDNA sequence of *OppLTL* was obtained by a transcriptome of the spotted knifejaw and confirmed by PCR and sequencing. The ORF of *OppLTL* was 354 bp, encoding 117 amino acids, and had a calculated molecular mass of 13.56 kDa and a theoretical pI of 7.73. The protein domain prediction showed that OppLTL had a conserved B-type lectin domain/CRD domain formed by residues 3–113 (**Figures 1A, D** **)**. The secondary structure of OppLTL illustrated that it contained 0.85% α-helix, 39.3% β-sheet, and 59.83% random coil. Furthermore, the whole OppLTL consisted of 12 β-sheets, which were located in three false triple symmetry subdomains. Each subdomain had four anti-parallel β-sheets. The D-mannose binding sites were located in the crocks of β-sheets in each subdomain (**Figures 1B** **, C**).

### Sequence and phylogenetic analysis

The multiple alignment analysis of the CRD domain of LTL showed that three D-mannose binding sites were found in different species. The first two motifs were highly conserved, whereas the third one was less conserved (**Supplementary Figure S1**). Three D-mannose binding sites were also found in the CRD domain of OppLTL in spotted knifejaw, which were in the forms of ^30^Q×D×N×V×Y^38^, ^59^Q×D×N×V×Y^67^, and ^92^T×D×K×V×Y^100^. The phylogenetic tree was constructed by neighbor-joining method to confirm the relationship among different species. The phylogenetic analysis indicated that the LTL homolog proteins could be divided into two distinct clades. The sequences of teleost and tetrapods were remarkably different and belonged to different groups (**Supplementary Figure S2**).

### Tissue distribution of *OppLTL* and challenge with *V. anguillarum*

**Figure 2:**
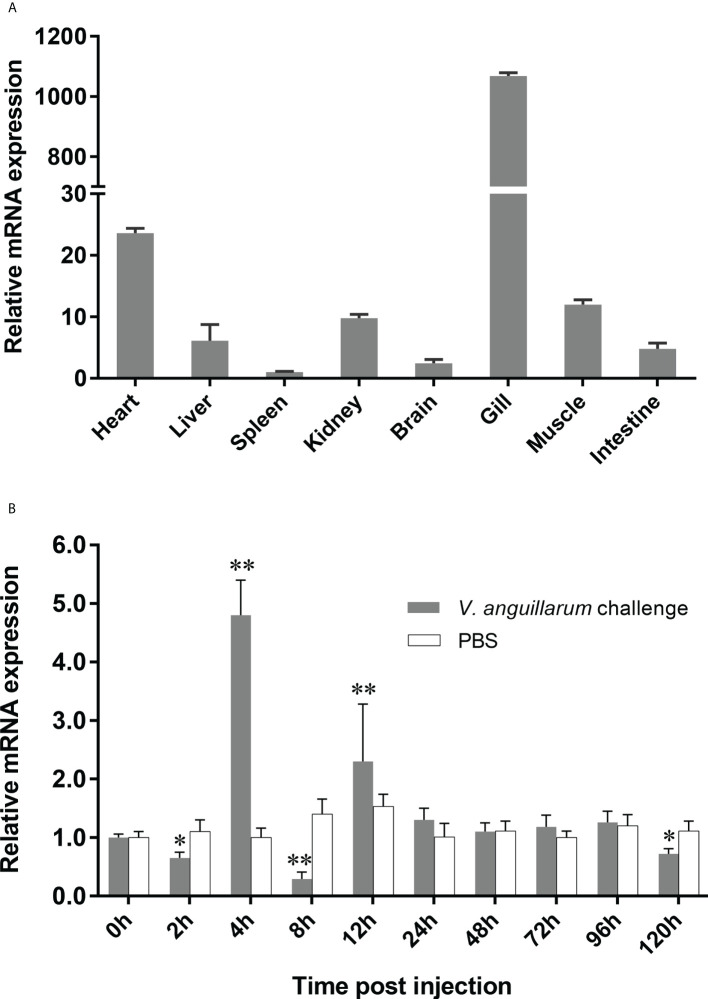
Expression profiles of *OppLTL* in normal tissues and when challenged with *V. anguillarum*. **(A)** Tissue distribution of the *OppLTL* expression level. **(B)** *OppLTL* expression in response to the *V. anguillarum* challenge in gill. Data are shown as mean ± SD (*n* = 3). Significant difference (*P < 0.05 and **P < 0.01) between *V. anguillarum*-challenged tissues and blank control (at 0 h) is indicated with an asterisk.

To detect the distribution of *OppLTL* in tissues, the expression of *OppLTL* was analyzed by qRT-PCR. *OppLTL* was expressed in all tested tissues but showed different levels. The *OppLTL* transcripts were highly expressed in the gill but weakly expressed in the heart, liver, spleen, kidney, brain, muscle, and intestine (**Figure 2A**). Given the specific expression of *OppLTL* in the gill, we examined the change of *OppLTL* in the gill at 2, 4, 8, 12, 24, 48, 72, 96, and 120 h after the *V. anguillarum* challenge. The results demonstrated that the expression of *OppLTL* was up- or downregulated remarkably at different time points. The first and second peak appeared at 4 and 12 h after injection, respectively (**Figure 2B**).

### Recombinant expression and purification of OppLTL

**Figure 3:**
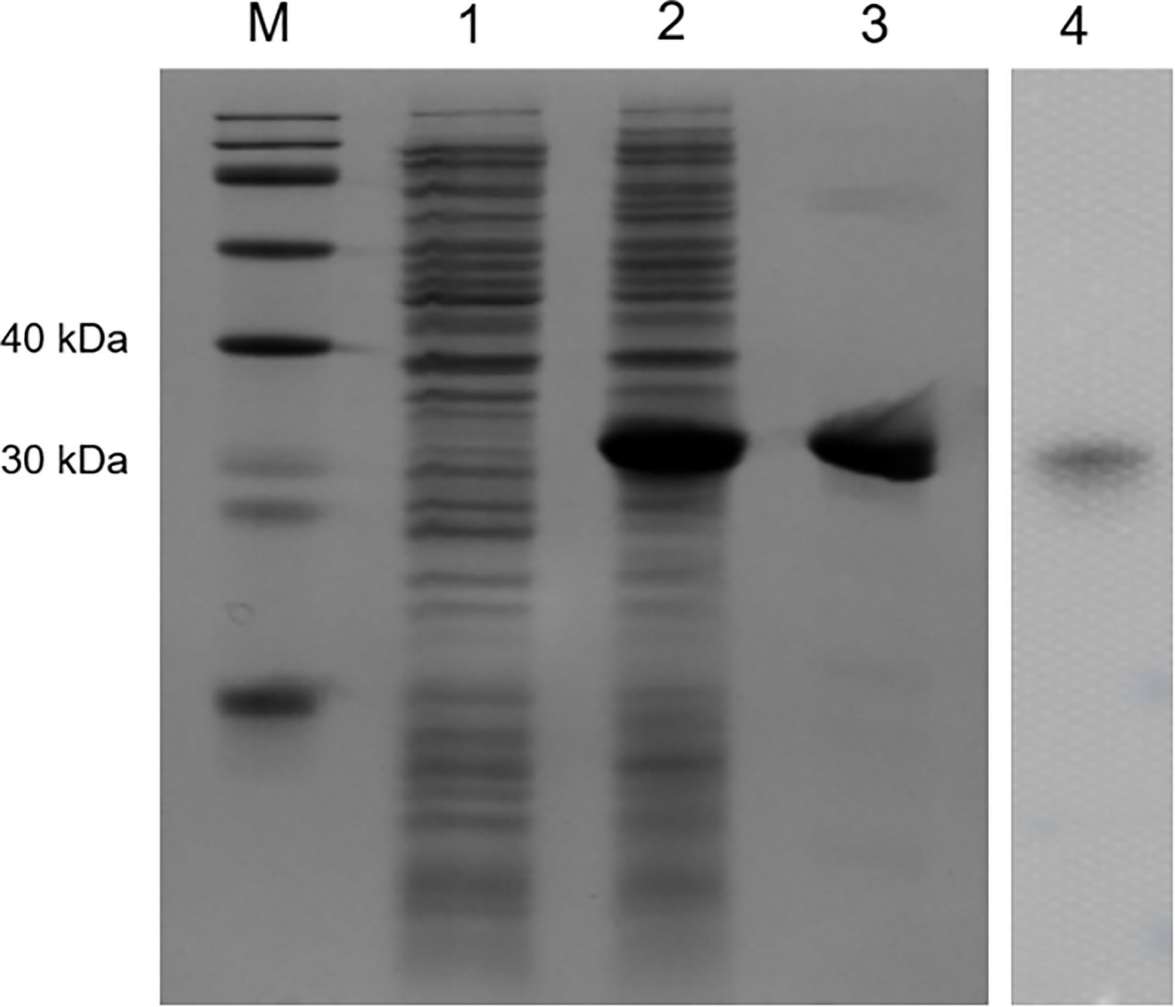
SDS-PAGE analysis and western blotting detection of recombinant protein rOppLTL. Lane M: marker; lane 1: extracts from expressing strains before isopropyl β-D-thiogalactopyranoside (IPTG) induction; lane 2: extracts from IPTG-induced expressing strains; lane 3: purified rOppLTL; lane 4: western blot of purified rOppLTL.

Recombinant OppLTL was expressed in *E. coli* BL21 and purified with glutathione resin. No signal peptide was predicted in OppLTL. The predicted molecular mass of OppLTL was approximately 17.7 kDa, and the rTRX tag was 13.6 kDa. rOppLTL (31.3 kDa) was consistent with the size of a major band that appeared in the purified protein lane. The concentration of rOppLTL was 1.02 mg/ml after being purified and refolded. The western blot analysis showed that the purified protein was the target recombinant protein (**Figure 3**).

### Microbial agglutination and hemagglutination assay of rOppLTL

**Figure 4:**
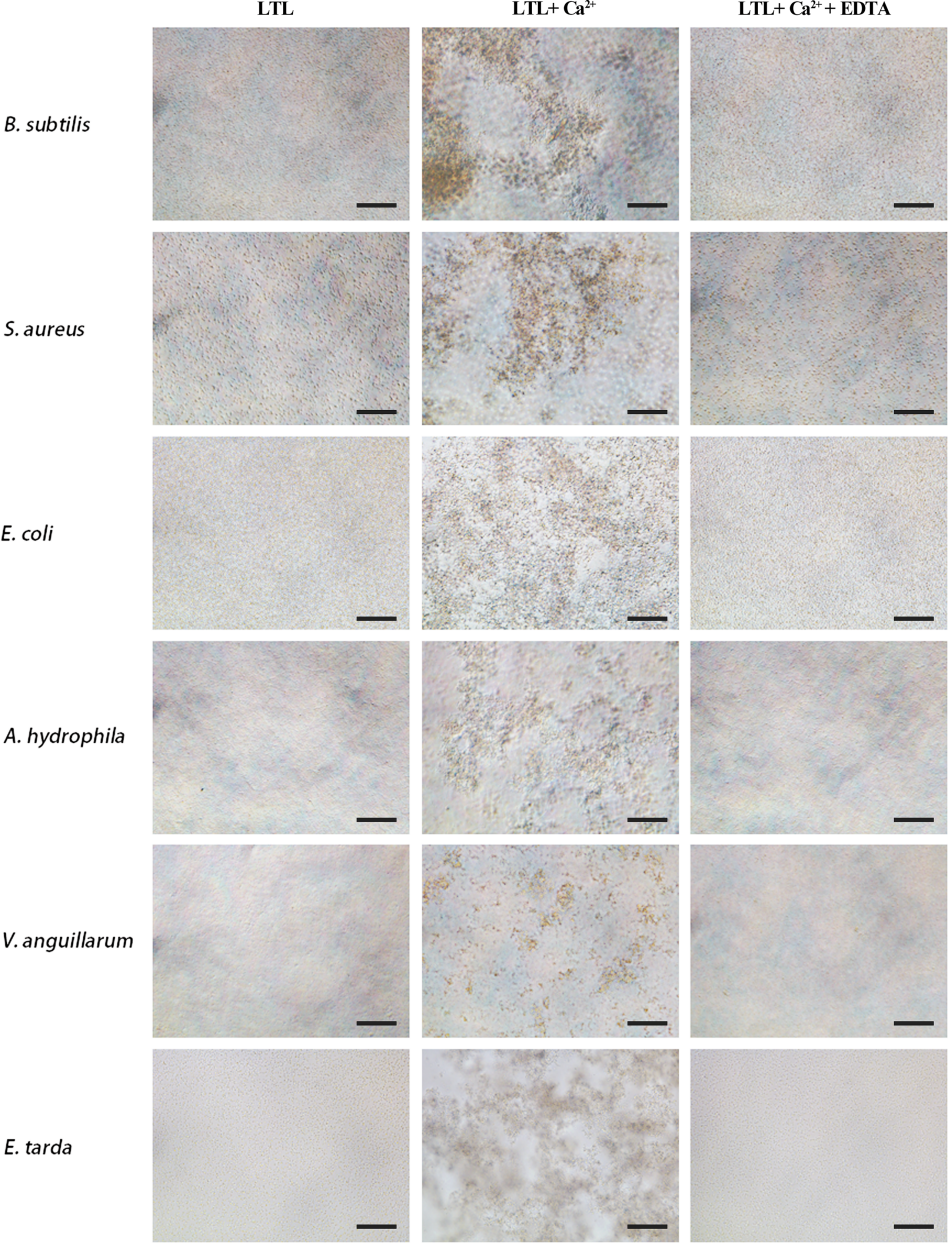
Agglutination activity of rOppLTL to bacteria. *B. subtilis*, *S. aureus*, *E coli*, *V. anguillarum*, *E tarda*, and *A. hydrophila* were incubated with rOppLTL in the absence or presence of Ca^2+^. The cells were observed with a microscope. Bar: 10 μm.

LTL possesses antimicrobial activities. Its agglutination activity was investigated by microbial agglutination assay. The results demonstrated that rOppLTL could cause a strong agglutination of Gram-positive and Gram-negative bacteria in the presence of Ca^2+^. Simultaneously, the agglutination activities completely disappeared in Ca^2+^-depleted groups (EDTA supplementary groups), indicating that agglutination occurred in a Ca^2+^-dependent manner (**Figure 4**).

**Figure 5:**
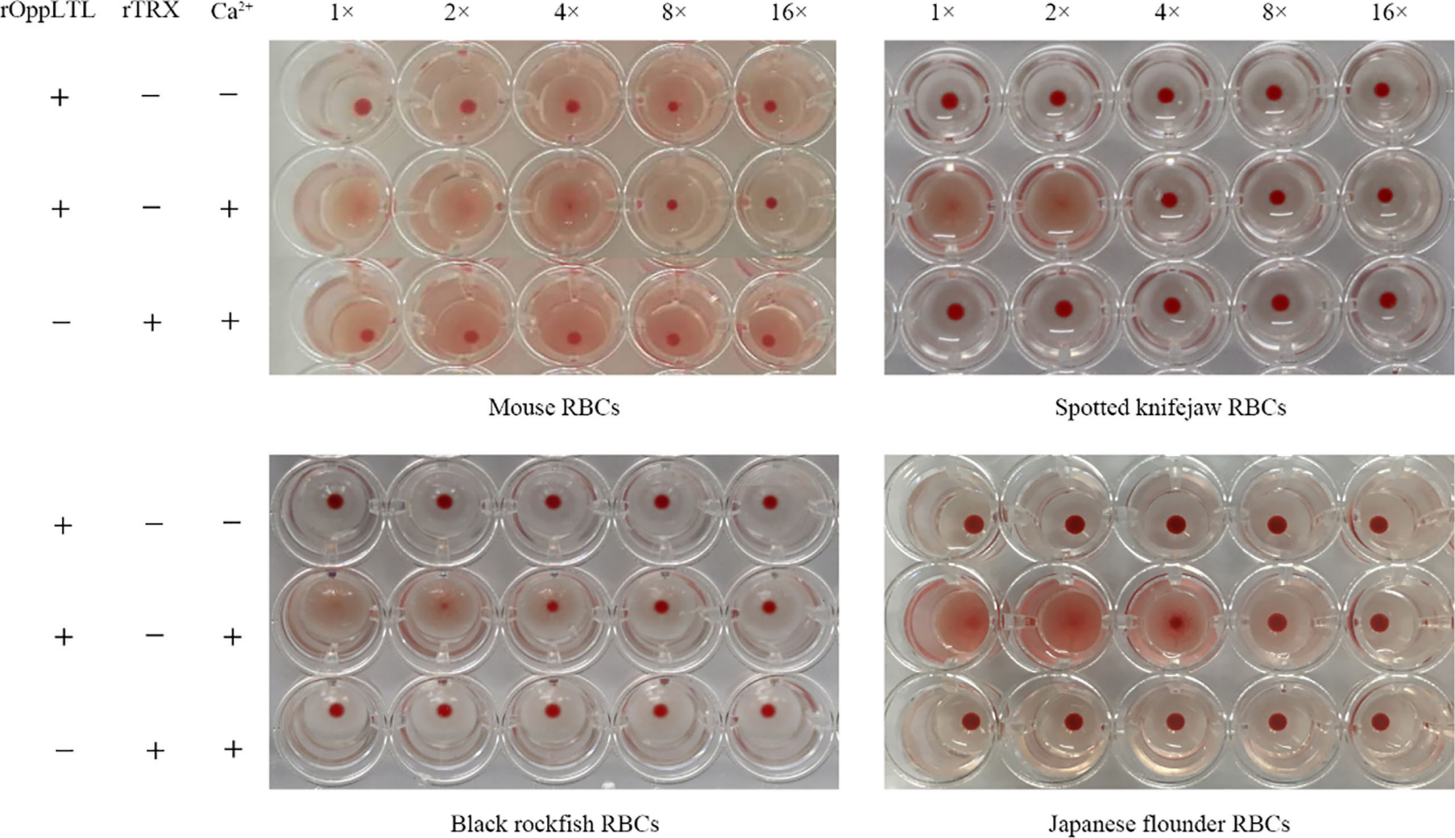
Agglutination activity of rOppLTL to red blood cells (RBCs). RBCs from mouse, spotted knifejaw, Japanese flounder, and black rockfish were incubated with rOppLTL and rTrx. The mixture was placed at room temperature for 1 h. "-" and "+" represent the absence and presence of rOppLTL, rTRX, and Ca2+, respectively.

The hemagglutination assay showed that rOppCTL could cause the agglutination of red blood cells (RBCs) from mouse, spotted knifejaw, Japanese flounder (*Paralichthys olivaceus*), and black rockfish in the presence of Ca^2+^ but not in the absence of calcium. Moreover, mouse RBCs appeared to be more sensitive to rOppCTL than others (**Figure 5**).

### Binding activity of rOppLTL

**Figure 6:**
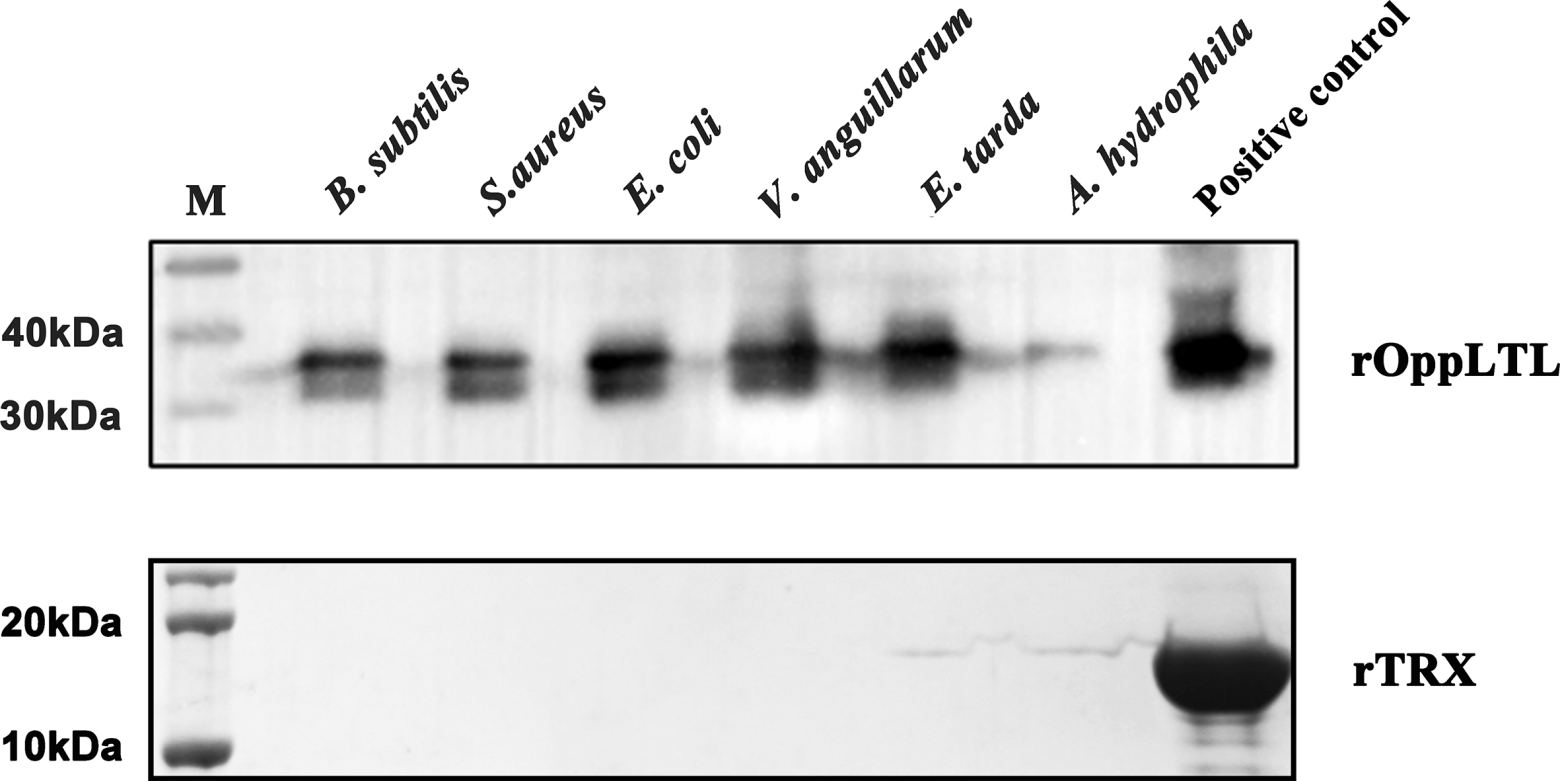
Binding activity of rOppCTL to microorganisms. Lane M: pre-stained marker (kDa); lane 1: *B subtilis*; lane 2: *S. aureus*; lane 3: *E coli*; lane 4: *A hydrophila*; lane 5: *V. anguillarum*; lane 6: *E tarda*; lane 7: rOppLTL/rTRX as positive control. The binding activity of rTRX protein was tested and used as the negative control.

To clarify the potential antibacterial mechanism of rOppLTL, a microorganism binding assay was carried out to analyze the ability of rOppLTL to bind pathogenic microbes. The results revealed that rOppLTL was firmly attached to the selected bacteria including *B. subtilis*, *S. aureus*, *E. coli*, *V. anguillarum*, *E. tarda*, and *A. hydrophila*. rOppLTL exhibited broad binding activities to these microorganisms, but the capability was different among bacteria. The binding activities of rOppLTL to bacteria were strong except for *A. hydrophila* (**Figure 6**).

**Figure 7:**
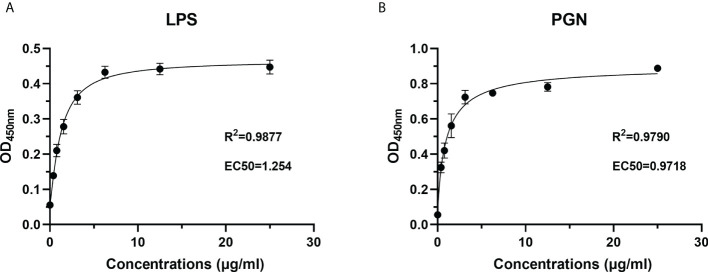
Recombinant protein rOppLTL binds to polysaccharides. **(A)** ELISA analysis of the binding ability of rOppLTL to LPS. **(B)** ELISA analysis of the binding ability of rOppLTL to PGN.

To characterize the PAMP recognition capacity of rOppLTL, ELISA was used to measure the binding ability of rOppLTL to LPS and PGN. Different concentrations (from 0 to 25 μg/ml) of recombinant protein were used during ELISA to evaluate the binding ability of rOppLTL. The results showed that rOppLTL exhibited binding affinity toward LPS (EC50 = 1.254 μg/ml) and PGN (EC50 = 0.9718 μg/ml) in a dose-dependent manner. The binding curve fits the logarithmic curve, showing that the binding between recombinant protein and polysaccharides was saturable (**Figure 7**). By contrast, rTRX (as a control) did not exhibit any binding activity to polysaccharides.

### Subcellular localization of OppLTL

**Figure 8:**
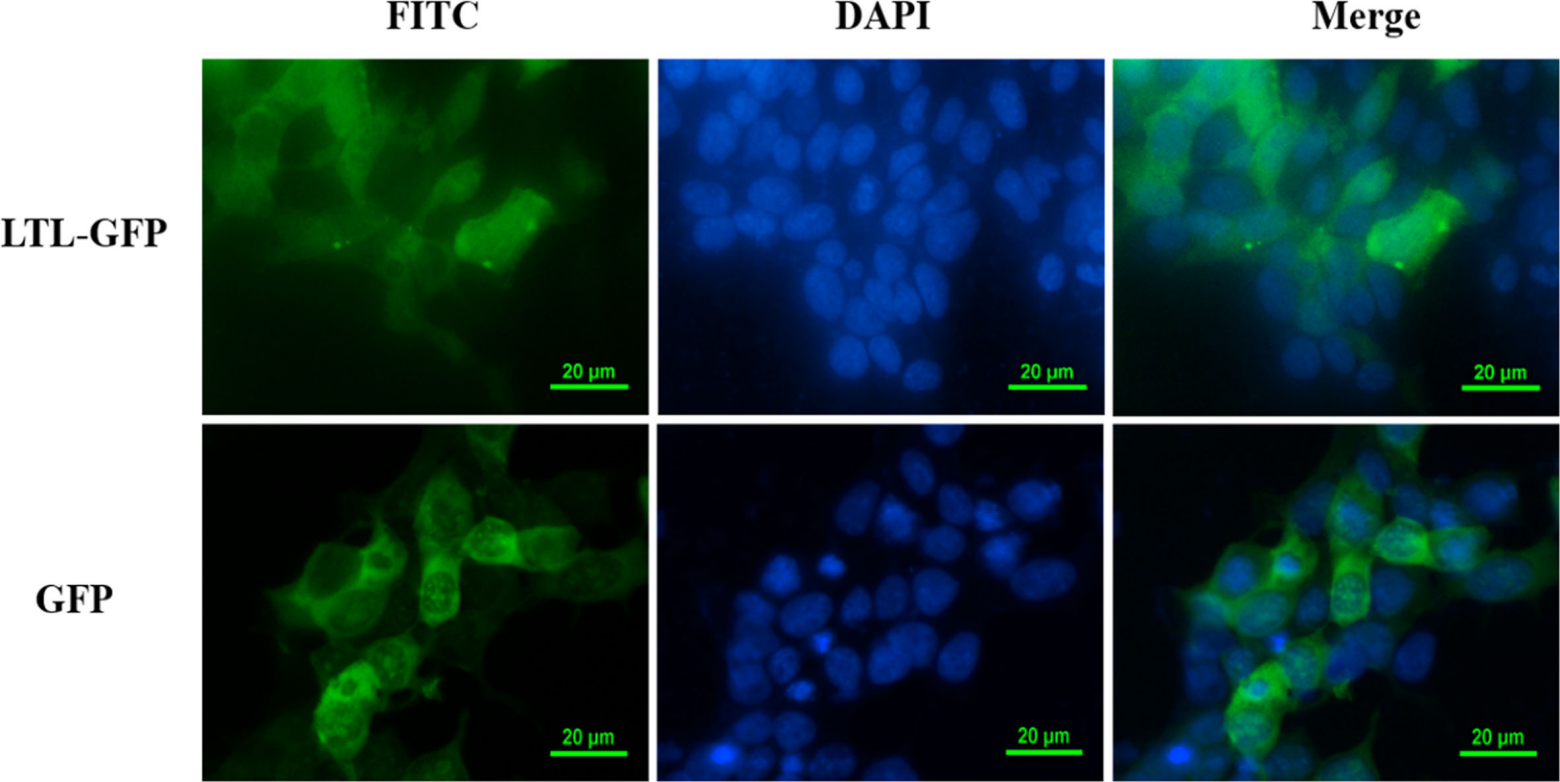
Subcellular localization analysis of OppLTL-EGFP fusion proteins expressed in HEK293T cells by transfection of OppLTL-EGFP plasmid fluorescence microscopy.

To further explore the function of OppLTL in spotted knifejaw, subcellular localization of OppLTL was identified in HEK293T cells. pEGFP-OppLTL was used for OppLTL localization. It was revealed that OppLTL was mainly distributed in the cytoplasm, while a little amount of signal was detected in the nucleus of HEK293T cells. By contrast, GFP was located in the nucleus and cytoplasm after pEGFP-N1 vector transfection (**Figure 8**).

### Inhibition of the NF-κB signal pathway mediated by OppCTL

**Figure 9:**
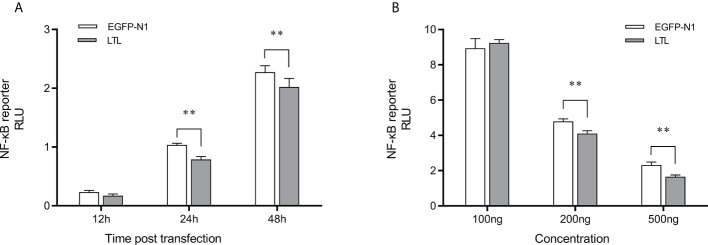
Effects of overexpressed OppLTL on the activation of the NF-κB signal pathway. **(A)** The 500-ng OppLTL-EGFP with 100 ng NF-κB luciferase reporter plasmids was co-transfected into HEK293T cells. Luciferase detection was applied at 12, 24, and 48 h after transfection, respectively. **(B)** Furthermore, 100, 200, and 500 of ng OppLTL-EGFP were co-transfected with 100 ng NF-κB luciferase reporter plasmids into HEK293T cells, respectively. Luciferase detection was applied at 48 h after transfection. Data was shown as mean ± SD (*n* = 6). Bars with an asterisk symbol indicate significant differences (**P < 0.01).

To assess the effects of OppLTL on the NF-κB signaling pathways, luciferase reporter gene assay was performed. The recombined pEGFP-OppLTL plasmid could significantly inhibit the activity of NF-κB luciferase reporters in HEK293T cells at 12, 24, and 48 h post-transfection compared with the empty vector pEGFP-N1 (**Figure 9A**). Besides this, OppLTL overexpression induced a dose-dependent inhibition of the NF-κB signal pathway in different concentrations (**Figure 9B**).

## Discussion

PRRs play important roles in pathogen identification and innate immune response. As PRRs, it has been reported that lectins serve as key components in the innate immune system by interacting with pathogens (26–29). In this study, *OppLTL* was identified and functionally analyzed from spotted knifejaw. *OppLTL* possesses a B-type mannose-specific lectin domain and three repeats of the conserved motif QXDXNXVXY, and the third repeat is not conserved. In previous studies, the conserved motif could bind D-mannose in plant B-type lectins (30, 31). The phylogenetic analysis showed that OppLTL was grouped closely with other teleost OppLTL, suggesting that LTL in teleost was evolutionarily conserved. The structure of OppLTL was similar to that in other species. Three D-mannose binding sites were also found in the OppLTL structure. In general, the gene structure and the amino acid sequence of OppLTL were conserved. According to the conservation of sequence and evolution, we speculated that OppLTL possessed the binding activity of D-mannose, and it was a microbial binding lectin.

qRT-PCR was performed to detect the tissue distribution pattern of *OppLTL*. *OppLTL* was expressed in all tested tissues, and it was highly expressed in the gill. In a previous study, the distribution of *LTL* was tissue specific in different species. *LTL-2* and *LTL-3* were highly expressed in the liver and skin of rock bream (10). A high expression of *LTL* was detected in the intestine, gill, and skin of black rockfish (14). In tongue sole, a high expression of three B-type lectins was observed in the liver and blood. In turbot, it was mainly expressed in the skin, intestine, and gill (13). In general, the *LTLs* of teleost were detected in tissues involved in immunity (9). These results suggested that *LTLs* not only played critical roles in the innate immune system in teleost. It might be involved in the adaptive immune system by mucosal immunity. Similar to other lectins in teleost, the expression level was upregulated by stimulation with bacterial pathogens (8, 32, 33). In this study, *OppLTL* expression was enhanced significantly in the gill after being challenged with bacterial pathogens. It was reported that *LTLs* were significantly induced after *E. tarda*, *V. harveyi*, and *Micrococcus luteus* infection in tongue sole (13). Besides this, *LTL* responded to viral infection after being challenged with RBIV in rock bream (10). Similar expression profiles were detected in the gill and blood of black rockfish after being challenged with LPS, Poly I:C, and *Streptococcus iniae* (14). The expression peaked early and then decreased to the baseline, followed by another increase at later time points. The fluctuation of the expression patterns might be ascribed to the homeostatic balance in the immune matrix. The inflammatory factors could be upregulated following the expression of LTL in the early period of bacterial infection. To avoid inflammatory storm, the expression of LTL decreased immediately and then increased slowly. Aside from pathogens, *LTL* from *Scophthalmus maximus* (named *SmLTL*) could be regulated by environmental factors, such as temperature and salinity. *SmLTL* was found to be upregulated in the gill, intestine, and skin when turbot was infected with ciliate (*Philasterides dicentrarchi*) (9). These results indicated that *OppLTL* was involved in immune response during a pathogenic infection. The increase of *OppLTL* might enhance the defense against bacteria or protozoa. Previous studies had reported the ability of lectins to interact with bacteria or viruses by recognizing the surface glycans in fish (4–6). Combining all the results of the present study and of the previous reports, it is reasonable to infer that *OppLTLs* play critical roles in the innate immunity of spotted knifejaw *via* recognition and agglutination of bacterial pathogens.

It was verified that most of the lectins played the function depending on Ca^2+^. C-type lectin (CTL) from spotted knifejaw was dependent on Ca^2+^- (22). LTLs from tongue sole and turbot were also dependent on Ca^2+^ (13). However, some lectins were Ca^2+^ independent, such as NTL in Qihe crucian carp (*Carassius auratus*), CTL-1 in amphioxus, and IML-2 in tobacco hornworm (*Manduca sexta*) (34–36). In our study, rOppLTL could bind all tested microorganisms in the presence of Ca^2+^. LPS and PGN were used to determine the binding ability of rOppLTL to the outer membranes of Gram-negative/positive bacteria. ELISA showed that rOPPLTL exhibited binding affinity in a dose- and Ca^2+^-dependent manner. Therefore, OppLTL is a Ca^2+^-dependent L-type lectin.

Studies indicated that lectins possess agglutination and hemagglutination abilities to bacteria or fungi (2, 8–10, 13). Lectins with bacteriostatic and bactericidal effects have been reported in plants (37–39). In teleost, a mannose-binding lectin displayed an antibacterial effect against *E. coli*, and GANL could inhibit the growth of *V. harveyi* (40, 41). In addition, some BMLs could kill target bacteria and inhibit bacterial dissemination in fish (13). In our study, rOppLTL was able to agglutinate multiple bacteria depending on Ca^2+^, but the agglutination effect was different. Meanwhile, the binding ability of rOppLTL to bacteria was not the same. The binding ability was weaker to *V. harveyi* than to other microorganisms. However, CTL showed a strong binding ability to *V. harveyi* in spotted knifejaw (22). The hemagglutination assays exhibited a positive effect on the hemagglutination of fish erythrocytes. Antibodies and other serum components could agglutinate pathogens. Teleosts lack antibody diversity as a primitive vertebrate. Lectins may supplement the poor secondary immune response and enhance the capacity of innate immune recognition. How lectins participate in immune response remain unknown. In plants, lectins from *Bryothamnion triquetrum* and *Lonchocarpus campestris* could inhibit inflammatory nociception and anti-inflammatory activity (42–45). In the present study, OppLTL inhibited the activity of the NF-κB signal pathway, suggesting its potential anti-inflammatory activity. Although the changes were low in percentage, it was significant in the statistical analysis. As we know, lectins were diverse in structure and function. They played roles such as recognition and effector factors in innate immunity and modulators of adaptive immune responses (2). We speculated that there might be several reasons to influence the inhibition efficiency. Firstly, it might be influenced by the concentration of OppLTL because our data indicated that the inhibition of the NF-κB signal pathway was dose dependent. Secondly, OppLTL could interact with the NF-κB signal pathway directly. Meanwhile, it might also interact with other pathways related to immune response, such as JAK/STAT, Toll-like, and PI3K-AKT signal pathways. Thirdly, the inhibition of OppLTL to the NF-κB signal pathway might be in coordination with the co-factor. The function of OppLTL was limited when it existed alone. The specific mechanism needs further study.

In conclusion, *OppLTL* was identified and characterized from spotted knifejaw. The mRNA of *OppLTL* was mainly expressed in the gill. The expression of *OppLTL* was remarkably upregulated following the bacterial challenge. rOppLTL displayed binding and agglutinating activities against Gram-positive and Gram-negative bacteria in a Ca^2+^-dependent manner. The subcellular localization revealed the OppLTL was mainly detected in the cytoplasm and nucleus. OppLTL could inhibit the NF-κB signal pathway as a potential immunosuppressive factor in anti-inflammatory reaction. Collectively, these findings indicated that OppLTL might be implicated in innate immunity through pattern recognition and pathogen elimination in spotted knifejaw.

## Data availability statement

The original contributions presented in the study are included in the article/**Supplementary Material**. Further inquiries can be directed to the corresponding author.

## Ethics statement

This research was conducted in accordance with the protocols of the Institutional Animal Care and Use Committee of the Ocean University of China.

## Author contributions

All authors listed have made a substantial, direct, and intellectual contribution to the work and approved it for publication.

## Funding

This research was supported by the Research Program of Sanya Yazhou Bay Science and Technology City (SKJC-2020-02-009, SKJC-KJ-2019KY01) and the National Natural Science Foundation of China (31802327).

## Conflict of interest

The authors declare that the research was conducted in the absence of any commercial or financial relationships that could be construed as a potential conflict of interest.

## Publisher’s note

All claims expressed in this article are solely those of the authors and do not necessarily represent those of their affiliated organizations, or those of the publisher, the editors and the reviewers. Any product that may be evaluated in this article, or claim that may be made by its manufacturer, is not guaranteed or endorsed by the publisher.

## Supplementary material

Supplementary Figure 1 Multiple alignment of B-lectin domain sequences from OppLTL and other fishes. The three conserved D-mannose binding sites are marked with blue boxes. The accession numbers are as follows: *Oplegnathus fasciatus* (ADV35591.1, ADV35592.1, and ADV35593.1), *Sebastes schlegelii* (ATS17370.1), *Larimichthys crocea* (ADN97105.1), *Nuchequula nuchalis* (BAE79275.1), *Platycephalus indicus* (BAE79274.1), *Epinephelus coioides* (AEG78370.1), *Channa striata* (CCQ25776.1 and CCQ48558.1), and *Scophthalmus maximus* (ANB44750.1).

Supplementary Figure 2 Construction of a phylogenetic tree with the protein sequences of LTL. The phylogenetic tree was constructed by MEGA X with neighbor-joining method based on multiple sequence alignment by Clustal W. The reliability of each node was estimated by bootstrapping with 1,000 replications. The numbers shown at each node indicate the bootstrap values (%).
