# Supplementary figures and images for: Immunological characterization and function analysis of L-type lectin from spotted knifejaw, Oplegnathus punctatus
Source: Front Immunol. 2022 Sep 26;13:993777. doi: 10.3389/fimmu.2022.993777 (PMC9549603; doi:10.3389/fimmu.2022.993777)

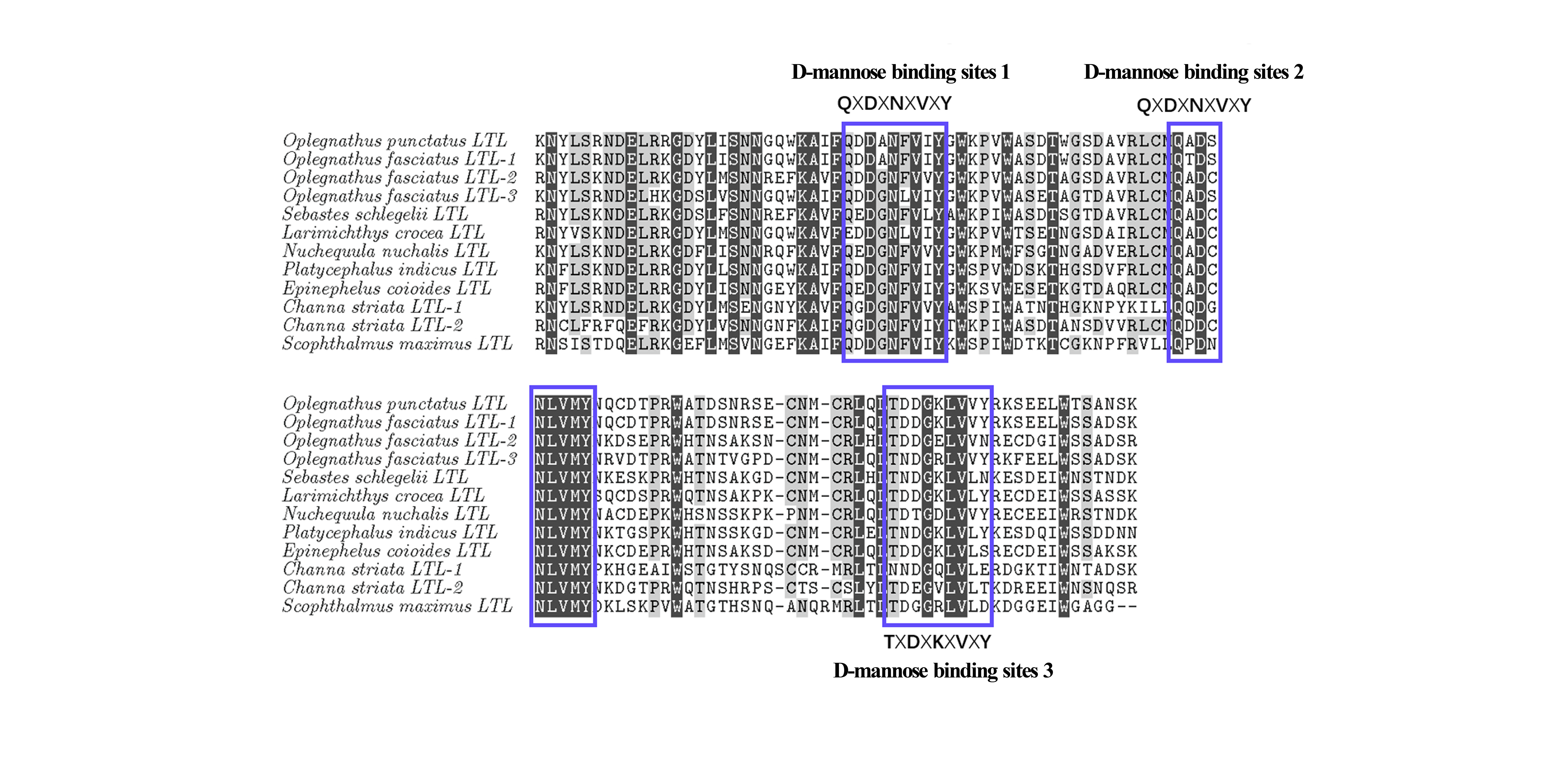

Supplement: Supplementary Figure 1 — Multiple alignment of B-lectin domain sequences from OppLTL and other fishes. The three conserved D-mannose binding sites are marked with blue boxes. The accession numbers are as follows: Oplegnathus fasciatus (ADV35591.1, ADV35592.1, and ADV35593.1), Sebastes schlegelii (ATS17370.1), Larimichthys crocea (ADN97105.1), Nuchequula nuchalis (BAE79275.1), Platycephalus indicus (BAE79274.1), Epinephelus coioides (AEG78370.1), Channa striata (CCQ25776.1 and CCQ48558.1), and Scophthalmus maximus (ANB44750.1). [file Image_1.tif]

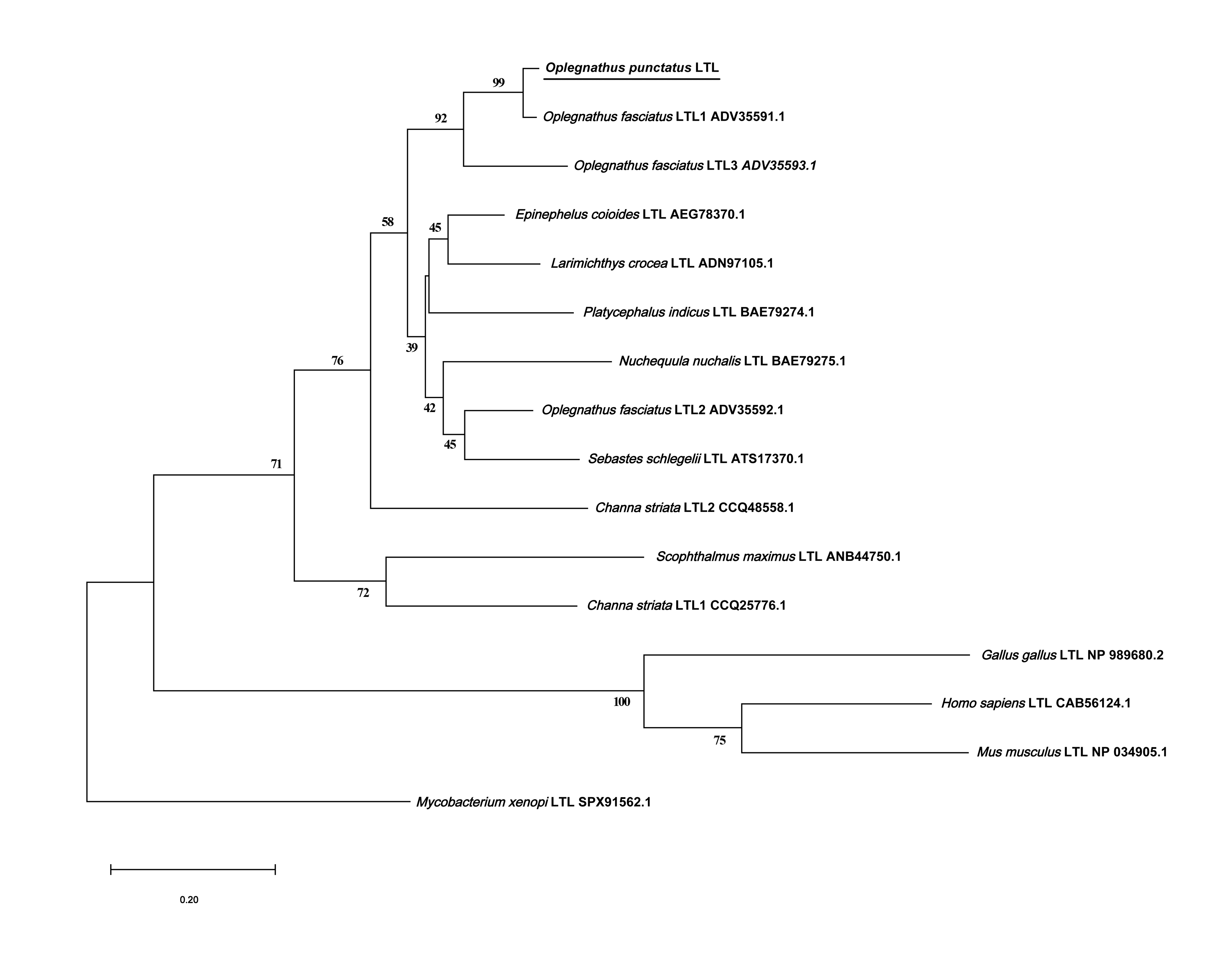

Supplement: Supplementary Figure 2 — Construction of a phylogenetic tree with the protein sequences of LTL. The phylogenetic tree was constructed by MEGA X with neighbor-joining method based on multiple sequence alignment by Clustal W. The reliability of each node was estimated by bootstrapping with 1,000 replications. The numbers shown at each node indicate the bootstrap values (%). [file Image_2.tif]
